# Review of the Molecular Genetics of Basal Cell Carcinoma; Inherited Susceptibility, Somatic Mutations, and Targeted Therapeutics

**DOI:** 10.3390/cancers13153870

**Published:** 2021-07-31

**Authors:** James M. Kilgour, Justin L. Jia, Kavita Y. Sarin

**Affiliations:** Department of Dermatology, Stanford University School of Medcine, Stanford, CA 94305, USA; jkilgour@stanford.edu (J.M.K.); jljia@stanford.edu (J.L.J.)

**Keywords:** basal cell carcinoma, skin neoplasms, dermatology, review, molecular genetics, germline mutation, somatic mutation, molecular targeted therapy

## Abstract

**Simple Summary:**

Basal cell carcinoma is the most common human cancer worldwide. The molecular basis of BCC involves an interplay of inherited genetic susceptibility and somatic mutations, commonly induced by exposure to UV radiation. In this review, we outline the currently known germline and somatic mutations implicated in the pathogenesis of BCC with particular attention paid toward affected molecular pathways. We also discuss polymorphisms and associated phenotypic traits in addition to active areas of BCC research. We finally provide a brief overview of existing non-surgical treatments and emerging targeted therapeutics for BCC such as Hedgehog pathway inhibitors, immune modulators, and histone deacetylase inhibitors.

**Abstract:**

Basal cell carcinoma (BCC) is a significant public health concern, with more than 3 million cases occurring each year in the United States, and with an increasing incidence. The molecular basis of BCC is complex, involving an interplay of inherited genetic susceptibility, including single nucleotide polymorphisms and genetic syndromes, and sporadic somatic mutations, often induced by carcinogenic exposure to UV radiation. This review outlines the currently known germline and somatic mutations implicated in the pathogenesis of BCC, including the key molecular pathways affected by these mutations, which drive oncogenesis. With advances in next generation sequencing and our understanding of the molecular genetics of BCC, established and emerging targeted therapeutics are offering new avenues for the non-surgical treatment of BCC. These agents, including Hedgehog pathway inhibitors, immune modulators, and histone deacetylase inhibitors, will also be discussed.

## 1. Introduction

Of all human cancers, basal cell carcinoma (BCC) is the most common worldwide, and in many countries, its incidence continues to increase, representing a significant public health burden [1]. Each year in the United States, more than 3 million cases occur, and one in five individuals in the U.S. are estimated to develop at least one BCC during their lifetime [2]. In addition to the potential morbidity of BCC, the cancer has a significant economic impact, with annual U.S. healthcare expenditure for these tumors reaching almost $4 billion [3,4,5]. The molecular pathogenesis of BCC is complex, and involves an interplay of inherited genetic susceptibility [6] and sporadic somatic mutations [7]. The former predisposes an individual towards the development of BCC, and can include single nucleotide polymorphisms (SNPs), inherited disorders, and genetic traits [6], but the latter is generally required to induce carcinogenesis. While the types of variants that induce susceptibility to BCC are varied, the sporadic mutations often function to activate the Hedgehog (HH) signaling pathway, a growth and development pathway integral to the pathogenesis of BCC. This review will discuss in more depth the currently known germline and somatic mutations implicated in the pathogenesis of BCC, as well as the underlying molecular pathways affected. We will also give an overview of established and emerging targeted, non-surgical, therapeutics which could revolutionize the treatment of this common and important skin cancer.

## 2. Inherited Susceptibility to BCC

Individuals can inherit an increased predisposition to BCC development, through genetic syndromes, germline SNPs and genetic traits [6]. Familial studies have been conducted to estimate the proportion of heritability of the keratinocyte-derived cancers (KC; i.e., BCC and squamous cell carcinoma). A study of 80,309 monozygotic and 123,382 dizygotic twins in Nordic countries reported a heritability of KC of 43% (95% confidence interval (CI) 26–59%) [8], while a study using data from the U.K. Biobank estimated that the heritability of BCC that could be explained by known inherited SNPs alone is 17% (95% CI 7–27%) [9].

### 2.1. Genetic Syndromes Associated with BCC Development

Some individuals face a much greater heritable risk of BCC than the general population, through inheritance of high penetrant germline mutations associated with one of 19 rare syndromes that have been linked to increased propensity of BCC. These syndromes, their associated mutations and molecular pathways, are outlined in Table 1 [6]. Research in the 1990s into the molecular basis of a rare genetic disorder known as Gorlin syndrome (also known as Nevoid Basal Cell Carcinoma syndrome) led to new understanding of the key oncogenic pathway underlying the etiology of BCC. Gorlin syndrome affects as many as 1 in 31,000 individuals in the U.K. [10], and is inherited through an autosomal dominant pattern with a high degree of penetrance but with variable phenotype [11]. Major clinical features of the syndrome include multiple, early-onset BCCs, jaw odontogenic keratocysts, palmar and plantar pits, and lamellar calcification of the falx cerebri [12]. BCCs present at a median age of 25, typically on sun exposed sites, and can range from few to thousands in number [13,14]. Interestingly, African American patients with Gorlin syndrome demonstrate less frequent development of BCC, reflecting the role of genetic factors such as pigmentation and epigenetic factors such as UV susceptibility in modulating carcinoma formation [14]. Similarly, SNPs in genes related to skin pigment, such as the melanocortin-1-receptor (*MC1R*) gene, have been associated with earlier and more severe onset of BCC [13].

The most frequent mutations attributed to causing Gorlin syndrome are germline loss of function mutations affecting *PTCH1* on chromosome 9q22.3 [15,16]. These inactivating mutations lead to premature termination of the PTCH protein, which is 7-transmembrane inhibitory receptor in the HH signaling pathway [17]. Interestingly, like the ocular cancer retinoblastoma, which similarly can be caused by inherited mutations in a recessive oncogene, a “two-hit hypothesis” has been proposed. This theory of carcinogenesis states that healthy cells require two separate mutagenic hits to produce carcinoma. Patients with inherited cancer syndromes, such as retinoblastoma and Gorlin, already have a preexisting germline mutation in one of their two copies of a tumor suppressor gene (i.e., *PTCH1*). This mutation alone is insufficient for cancer to occur. A second somatic mutation, as a result of UV radiation exposure for example, may subsequently induce malignancy through loss of the second copy of the tumor suppressor gene [18]. Other less commonly mutated genes implicated in Gorlin syndrome include *PTCH2* and *SUFU* [15].

**Figure 1:**
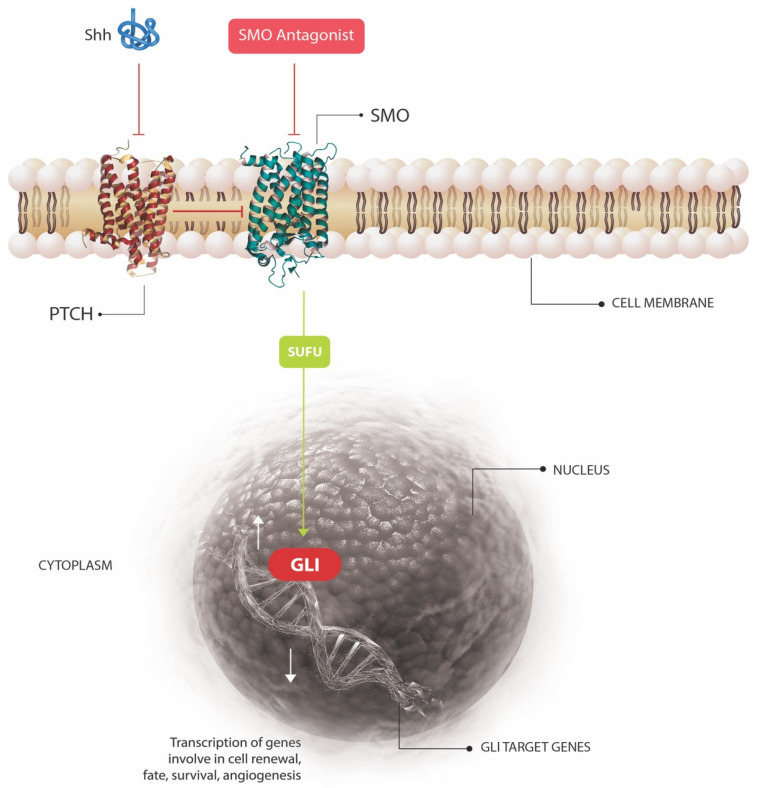
Overview of the Sonic Hedgehog signaling pathway. The HH ligand binds to a transmembrane receptor complex formed by the proteins PTCH and Smoothened (SMO). When not bound by the HH ligand, PTCH acts as a regulatory molecule, inhibiting translocation of SMO cilia thereby reducing HH signaling. When HH ligand binds to PTCH, the HH-PTCH complex is degraded by lysosomes which de-represses SMO, upregulating the pathway’s downstream signaling cascade via several proteins including suppressor of fused (SUFU). This ultimately results in release of members of the GLI protein family, such as GLI1, which are ordinarily sequestered in the cytoplasm [7,19]. GLI1 acts as a transcription factor, which upon release can translocate to the cell nucleus and trigger transcription of genes involved in cell renewal, fate, and survival, as well as angiogenesis [7].

The Sonic HH signaling pathway is an embryologically conserved pathway that is vital for determining tissue patterning and cell fate during embryo development [19]. Within the skin, the HH pathway is responsible for stem cell maintenance and developmental control of the hair follicles and sebaceous glands [7]. HH signaling is activated when the HH ligand binds to a transmembrane receptor complex formed by the proteins PTCH and Smoothened (SMO). When not bound by the HH ligand, PTCH acts as a regulatory molecule, inhibiting translocation of SMO thereby reducing HH signaling. When HH ligand binds to PTCH, the HH-PTCH complex is degraded by lysosomes which de-represses SMO, upregulating the pathway’s downstream signaling cascade via several proteins including suppressor of fused (SUFU). The ultimate result of this cascade is the release of members of the GLI protein family, which are ordinarily sequestered in the cytoplasm [7,19]. GLI acts as a transcription factor, which upon release can translocate to the cell nucleus and trigger transcription of genes involved in cell renewal, fate, and survival, as well as angiogenesis [7]. The normal functioning of the HH pathway is outlined in Figure 1. In Gorlin syndrome, germline inactivation of one copy of the *PTCH1* gene followed by somatic loss of the second allele result in loss of SMO suppression and hence constitutive overexpression of the HH signal. The downstream effect is overproduction of the GLI1 transcription factor, which acts to drive BCC tumorigenesis [18,20,21].

Bazex-Dupre-Christol syndrome is another genetic syndrome associated with the HH pathway and the development of multiple BCCs, alongside congenital hypotrichosis, follicular atrophoderma and milia [22]. It is inherited through an X-linked dominant pattern, with mutations in the *ACTRT1* gene most commonly implicated. These mutations lead to premature truncation of the ARP-T1 protein, which normally functions as an inhibitor of the GLI1 transcription factor through function at the *GLI1* gene promotor site. The mutation in *ACTRT1* consequently results in enhanced *GLI1*-induced oncogenic transcription [23].

In addition to germline defects in the HH signaling pathway, BCC susceptibility may occur due to inherited deficiencies in DNA repair. Xeroderma Pigmentosum (XP) is autosomal recessive disorder caused by inherited mutations in any one of eight possible genes required for nucleotide excision repair (NER). NER is the process by which NER endonucleases repair segments of DNA containing pyrimidine dimers, the major signature of UV-induced DNA damage characterized by covalent bonding of adjacent thymine nucleotides. Without repair, the shape of the DNA strand becomes disrupted. Consequently, XP patients have a significant UV-induced mutational burden, with strict lifelong avoidance of UV necessary for the prevention of skin cancer, including BCC, squamous cell carcinoma (SCC) and melanoma [24,25].

### 2.2. Certain Inherited Phenotypic Traits Modulate BCC Susceptibility

In addition to genetic disorders, certain inherited phenotypic traits have a demonstrated association with BCC risk. The traits associated with increased BCC formation are those pertaining principally to a fair skin phototype, and hence greater susceptibility to UV radiation-induced carcinogenesis. Individuals with fair skin, light-colored eyes and hair, childhood freckling, inability to tan and northern European ancestry are known to have increased rates of BCC development [26,27]. A meta-analysis of 29 studies by Khalesi et al. (2013) revealed that red hair, fair skin, and skin prone to burning and not tanning, is associated with a two-fold increased risk of BCC [28].

### 2.3. Gene Polymorphisms Associated with Modulated BCC Risk

Genome-wide association studies (GWAS) conducted in European populations in the U.S. and Iceland have identified 33 loci associated with BCC susceptibility [29,30,31,32,33,34,35,36]. Together, these 33 loci account for 10.98% of the heritability of BCC (Table 2). Many of these loci associated with altered BCC risk are shared with the other common KC, SCC [6]. Here, we will review in more depth the key biological pathways known to be implicated in BCC pathogenesis through the presence of gene polymorphisms.

### 2.4. Gene polymorphisms Affecting pigmentary traits

Traits associated with increased susceptibility to UV-damage, such as fair skin and light-colored eyes, are known to increase BCC risk. It is therefore not surprising that polymorphisms affecting genes involved in pigmentation modulate BCC predisposition. The melanocortin-1 receptor (*MC1R*) gene is one example; it codes for a transmembrane, G-protein coupled receptor that activates adenylate cyclase to produce intracellular cAMP in response to stimulation by α-melanocyte stimulating hormone (α-MSH). Signaling through cAMP in turn induces maturation of phenomelanosomes into eumelanosomes, which are responsible for darker pigmentation and therefore increased UV resistance [37]. The *MC1R* gene is highly polymorphic within light skinned individuals [37], and studies of common variants have identified significantly increased risk of BCC with certain of these variants, even after controlling for skin phototype and hair color, suggesting that it may confer increased risk through mechanisms independent of pigmentation [37,38]. Han et al. (2006) conducted a nested case–control study, reporting that the 151 Cysteine variant specifically was associated with an adjusted odds ratio (OR) of 1.56 for BCC (95% CI 1.03–2.34) [37]. Gudbjartsson et al. (2008) also identified polymorphisms within the TYR and ASIP pigmentation genes as significantly increasing BCC risk, with ORs of 1.14 (*p* = 6.1 × 10^−4^) and 1.35 (*p* = 1.2 × 10^−6^), respectively [39]. TYR encodes the enzyme tyrosinase, responsible for the production of melanin through the oxidation of tyrosine. *ASIP* codes for agouti signaling protein, which is an antagonist of the α-MSH and melanocortin-1 receptor interaction, therefore functioning to suppress melanin production [39]. GWAS studies have further identified risk loci adjacent to other pigmentation genes, including *IRF4, HERC2, LPP, BNC2, EXOC2, RALY* and *SLC45A2*, as being significant for BCC. While most of these loci are associated with increased risk, *SLC45A2, BNC2* and *HERC2* were all reported as having ORs of less than one, supporting decreased risk [29].

### 2.5. Gene Polymorphisms Affecting Tumor Suppressor Proteins

A polymorphism, rs78378222, in *TP53*, which encodes the tumor suppressor TP53 gene, is highly significant for BCC, with an overall OR of 2.16 (95% CI 1.83–2.54; *p* = 2.2 × 10^−20^). P53 can suppress tumor formation through enhancing the function of the protein p21, an inhibitor of the cell cycle regulator, cyclin D kinase. P53 also enhances apoptosis, via inhibition of BCL2, an anti-apoptotic protein [40], and also suppresses the HH pathway protein SMO [7]. The rs78378222 polymorphism affects the AATAAA polyadenylation signal of the 3′ untranslated region of the *TP53* gene, changing it to AATACA. This results in an impaired poly-adenine tail of the TP53 mRNA, which is necessary for stabilization and nuclear export [35]. Another important group of tumor suppressor genes are the cyclin D kinase inhibitor genes, *CDKN2A* and *CDKN2B*, which regulate the cell cycle. Stacey et al. (2009) identified that the rs2151280 polymorphism at the 9p21 loci near these genes is associated was an OR of 1.19 (95% CI 1.12–1.26; *p* = 6.9 × 10^−9^) for BCC [36].

### 2.6. Gene Polymorphisms Affecting Epidermal Differentiation and Cytoskeleton Organization

Genes determining epidermal differentiation and cytoskeleton organization have also been identified as harboring polymorphisms associated with increased BCC susceptibility. The *KRT5* gene produces the protein K5, which along with its heterodimeric partner K14, form the major keratins of the intermediate filament cytoskeletal network within basal keratinocytes. This network is vital for the basal layer’s structural integrity. The rs11170164 polymorphism results in a G138E substitution in the *KRT5* gene, resulting in an increased risk of BCC (OR 1.35; 95% CI 1.23–1.50; *p* = 2.1 × 10^−9^) [36]. Stacey et al. (2008) further identified the 1p36 and 1q42 loci as being risk-conferring for BCC, both of which contain genes involved in regulation of the cytoskeleton and keratinocyte differentiation [31].

### 2.7. Gene Polymorphisms Affecting NOTCH Signaling

**Figure 2:**
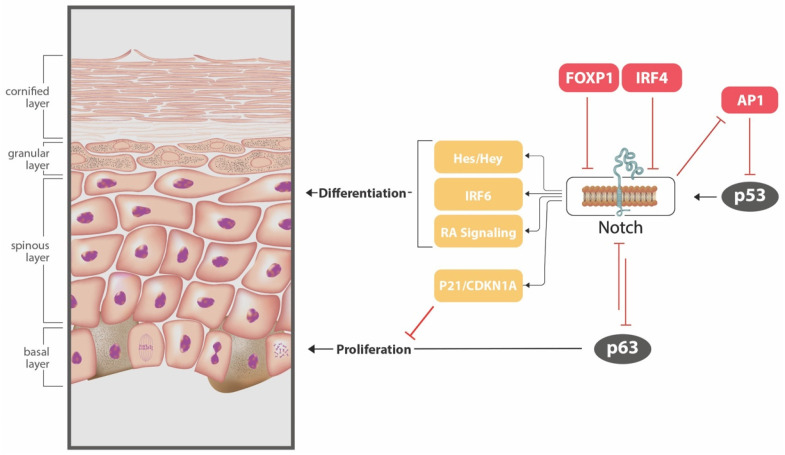
Downstream regulatory effects of NOTCH signaling on keratinocyte proliferation and differentiation. P53 induces NOTCH signaling though binding of a promotor region. Activation of the NOTCH signaling pathway involves proteolytic cleavage of the NOTCH intracellular domain, which is able to suppress AP-1 (a P53 inhibitor) further promoting NOTCH signaling. NOTCH down regulates keratinocyte proliferation through inhibition of p63, a transcription factor necessary for epidermal growth, and via enhancing expression of CDKN1A. NOTCH signaling regulates keratinocyte differentiation, through enhancing expression of transcription regulators (*IRF6* and the *Hes/Hey* genes), as well as promoting signaling through the retinoic acid pathway. *FOXP1* and *IRF4* play a regulatory role in NOTCH signaling, both having a repressive function.

The NOTCH signaling pathway has an important role in the regulation of keratinocyte proliferation and differentiation, and aberrant signaling through the pathway is known to be associated with cutaneous abnormalities and skin cancer [41]. In experiments of human nodular BCCs, NOTCH signaling has been shown to be depressed, and when treated with a Notch signaling peptide, increased apoptosis of BCC cells was revealed [42]. NOTCH signaling regulates epidermal development through a multitude of means and is complex (Figure 2). P53 is believed to induce NOTCH signaling, which in turn suppresses AP-1 (an p53 inhibitor), further promoting NOTCH. NOTCH signaling down regulates keratinocyte proliferation through two mechanisms: firstly, via inhibiting p63, a transcription factor necessary for epidermal growth, and secondly, via enhancing the expression of CDKN1A, a cell-cycle inhibitor. NOTCH signaling also regulates keratinocyte differentiation, through enhancing expression of transcription regulators such as *IRF6* and the *Hes/Hey* genes, as well as promoting signaling through the retinoic acid pathway via increased expression of various retinol-binding proteins [41]. Polymorphisms in regions of transcription factor genes (*FOXP1* and *IRF4*) which repress the NOTCH pathway have been implicated in increased propensity to BCC. The rs2116709 polymorphism of *FOXP1* is protective against BCC, with an OR of 0.90 (*p* = 2.3 × 10^−17^), while the rs12203592 variant of *IRF4* results in an increased risk of BCC (OR 1.48; *p* = 2.4 × 10^−152^) [29].

### 2.8. Gene Polymorphisms Affecting Telomere Maintenance

Instability of chromosomes is a known risk factor for KC, including BCC, as evidenced by predisposition to BCC as a feature of several inherited genetic disorders characterized by diminished chromosomal stability (Table 1) [6]. Chromosomal instability is believed to contribute to cancer pathogenesis through genetic variation that increases tumor adaptations that augment tumor survival [43]. Further, polymorphisms in genes related to telomere maintenance have been identified in several studies as associated with BCC risk. This includes variants affecting the gene *TERT*, which codes for the enzyme telomerase which adds protective repeat sequences to telomeres [33,36], and the gene *OBFC1*, a telomere-length regulating gene [29]. Increasing telomere length immortalizes cells, preventing senescence and permitting excessive cell replication, a feature of 90% of solid malignancies [44].

### 2.9. Gene Polymorphisms Affecting DNA Repair

UV radiation-induced DNA damage is a key carcinogenic mechanism in the pathogenesis of BCC, and the capacity for DNA repair genes to rectify this damage is important in preventing malignancy. Lin et al. studied SNPs across 165 DNA repair pathway genes, identifying variants within three key repair genes, *XPA, MUS81* and *NABP2*, which are all associated with significantly modulated risk of BCC. The former was reported to decrease propensity to BCC (OR 0.93), while the latter two increase the risk (ORs 1.06 and 1.11, respectively) [45].

### 2.10. Gene Polymorphisms Affecting Cutaneous Immunity

Cutaneous immunity and inflammation are known to modulate the risk of skin cancers, including BCC. Individuals with immunosuppression, such as those receiving solid organ transplants are known to suffer with a high burden of cutaneous malignancies, in part due to the loss of immune regulation and its anti-cancer effect. Polymorphisms within the human leukocyte antigen (HLA) region have been associated with altered BCC susceptibility, in addition to *IRF4* which has a role in immune regulation, and *UBAC2*, which is involved in inflammation [6]. Welsh et al. (2009) further studied the CT60 GG genotype of the cytotoxic lymphocyte-associated antigen-4 (*CTLA-4*) gene in a population-based cohort study of white individuals in New Hampshire. They reported that individuals with the CT60 GG genotype had decreased risk of BCC (OR 0.7; 95% CI 0.5–0.9). CTLA-4 plays an instrumental role in UV radiation-induced cutaneous immunosuppression, as it is expressed on t-regulatory cells that are up regulated by UV. CTLA-4 on t-regulatory cells interacts with antigen-presenting cells, suppressing t-lymphocyte activation and neutralizing the anti-tumor effect of effector t-lymphocytes. The CT60 GG genotype results in reduced t-regulatory cell function, and hence diminished UV-induced immunosuppression and ultimately augmented anti-tumor capacity. At the same time, individuals with this genotype are at amplified risk of multiple autoimmune diseases [46].

### 2.11. Gene Polymorphisms Associated with a Truncal, Clustering BCC Phenotype

In addition to modulating BCC risk, gene polymorphisms have also been associated with distinct phenotypes of BCC. One such phenotype is a constellation of younger male patients with clusters of multiple BCCs affecting non-sun exposed truncal skin [47]. This phenotype has been linked to germline polymorphisms of genes coding for the hepatic detoxification enzymes cytochrome p450 2D6 and glutathione s-transferase [48,49,50], as well as of the vitamin D receptor [50], and TNF-α [50,51].

## 3. Somatic Mutations Implicated in BCC Tumorigenesis

**Figure 3:**
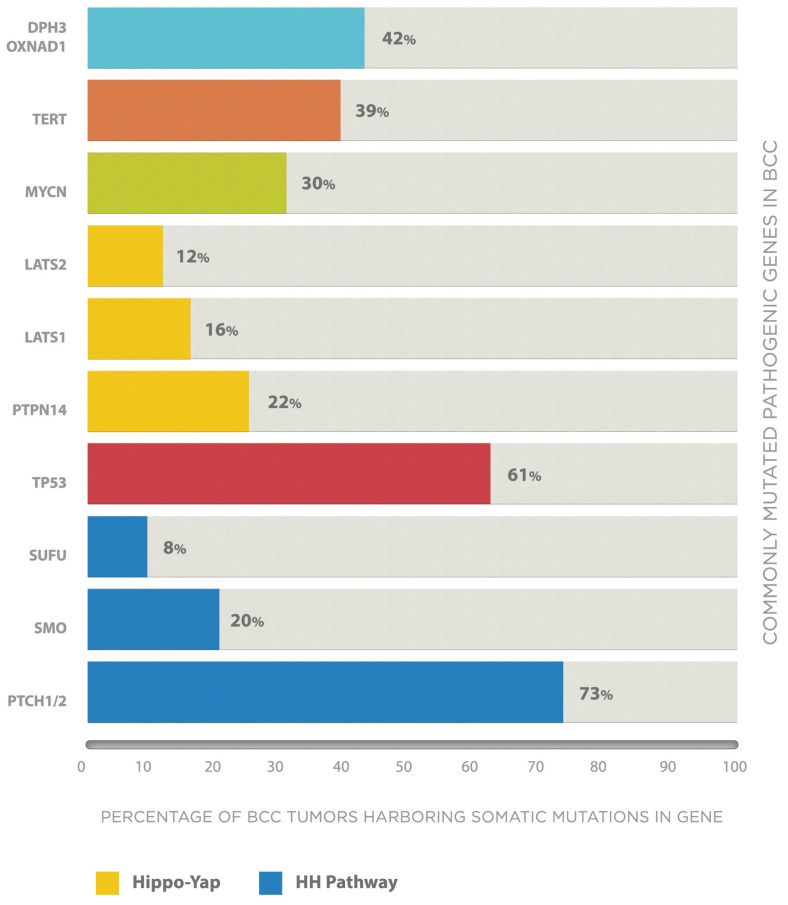
Cancer-related genes commonly affected by somatic mutations in BCC. Bar chart illustrates the common cancer-related genes in BCC affected by somatic mutations, with the percent of BCC tumors harboring such mutations as reported in genetic analysis studies. Genes are grouped by their respective molecular pathway, with genes coding for proteins in the HH pathway illustrated in blue, and the Hippo-Yap pathway in yellow. Percentages are taken from the studies with the largest sample size to date.

While germline polymorphisms result in an inherited predisposition to BCC, subsequent sporadic somatic mutations are required before cancer develops. Genomic analysis has found that BCC tumors are the most mutated of any human cancer, with on average 65 mutations per megabase [52], and the common cancer-related genes mutated in BCC are illustrated in Figure 3. The primary driver of the great mutational burden in BCC, and therefore the primary environmental risk factor, is exposure to UV radiation [53]. Ninety percent of single-nucleotide variants in a genomic analysis study were identified as mutations characteristic of UV-induced DNA damage (known as UV signature mutations), which is greater than that of other cutaneous malignancies such as melanoma [52].

**Figure 4:**
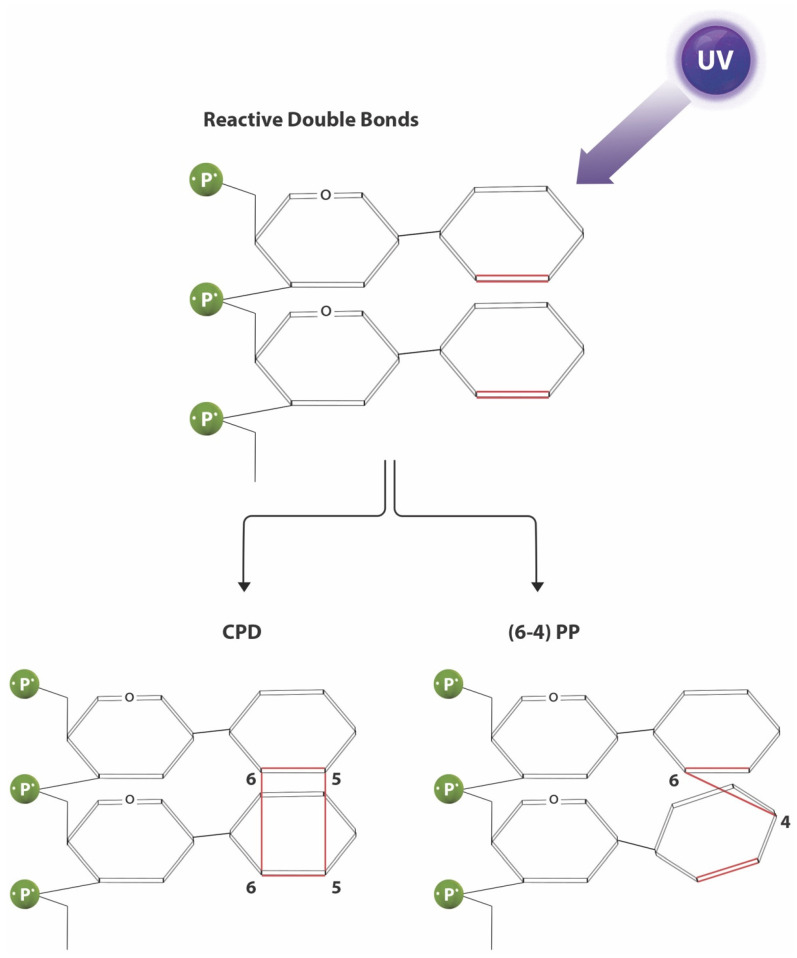
Photoproducts of direct UV-induced DNA damage. Absorption of UV radiation (principally UVB) by DNA strands result in the formation of photoproducts which disrupt the strand’s backbone, resulting in interruption of transcription and replication and the development of UV signature mutations. Cyclobutane pyrimidine dimers (CPDs) are characterized by formation of a cyclobutane ring by two adjacent thymine or cytosine bases. 6-pyrimidine-4-pyrimidone (6-4 PPs) photoproducts are formed by a single bond between the 6 carbon of the 5′ pyrimidine base and the 4 carbon of the 3′ pyrimidine base.

In particular, UV radiation is able to induce carcinogenesis in a multifaceted manner, through direct DNA damage, particularly involving oncogenes and tumor suppressor genes such as *PTCH1* and *p53* as previously discussed, suppression of cutaneous immunity and skin inflammation [6]. UVB is known to be highly mutagenic, as UVB is directly absorbed by DNA strands, resulting in UV signature mutations [54]. The two commonly identified DNA photoproducts generated primarily by UVB radiation absorption are cyclobutane pyrimidine dimers (CPDs) and 6-pyrimidine-4-pyrimidone (6-4 PPs) photoproducts (Figure 4). Both photoproducts cause disruption in the shape of the DNA strand, by distorting the strand’s backbone. Consequently, DNA transcription and replication by polymerases is interrupted, as these lesions act as stop points for the polymerases. Without repair, these lesions will induce UV signature mutations, defined as cytosine (C) to guanine (G) base substitutions and CC to GG tandem mutations. In addition to UVB, UVA may also be able to directly produce CPDs [54].

### 3.1. Frequent Somatic Mutations Identified in BCC: The HH Pathway

At least 90% of BCCs show enhanced signaling through the HH pathway through somatic loss of functions mutations in the *PTCH1* gene and activating mutations of SMO. The downstream consequence of these mutations is activation of the transcription factor GLI1 (Figure 1). The frequency of somatic *PTCH1* mutations in BCC studies is as high as 75% [7], and the mutations are typically non-synonymous, nonsense and splice site mutations found throughout the length of the gene. Supporting the important causative role of UV in BCC pathogenesis, 50% of *PTCH1* mutations are UV-signature in nature [7]. Activating mutations of SMO are similarly reported in 10–20% of BCCs [52], while less common HH pathway mutations include loss of function variants of SUFU (approximately 8% of BCCs) [52] and mutations in the *PTCH1*-homologue *PTCH2* [55].

### 3.2. TP53

The role of the essential tumor suppressor protein p53, coded for by the *TP53* gene, was outlined in our discussion of common germline polymorphisms associated with inherited susceptibility to BCC. Somatic mutations in *TP53* are frequent in all human cancers, and in BCC, non-synonymous mutations occur sporadically in approximately 61% of cases [7,52], with hotspots identified at codons 177, 196 and 245 [56,57]. Similarly to *PTCH1*, the majority of mutations in *TP53* in BCC are UV-signature [7].

### 3.3. The Hippo-Yap Signaling Pathway

The Hippo pathway is essential in tissue growth restriction, comprising of a cascade of kinases that use phosphorylation to suppress a downstream transcriptional co-activator, Yes-associated protein (YAP) [7]. Dysfunctional regulation of this pathway has been reported in BCC from RNA sequencing studies, with up-regulated YAP triggering basal keratinocyte proliferation [58]. Premature stop mutations of the *LATS1* gene, coding for one of the kinases in the Hippo pathway, have been reported in 16% of BCCs, and similarly, 23% of BCCs contain loss of function mutations in the *PTPN14* gene, a tumor suppressor functioning as a negative regulator of YAP. Furthermore, 12% of tumors may contain mutations in the *LATS2* gene, an analogue of LATS1 [52].

### 3.4. MYCN/FBXW7 Signaling

*MYCN* is a transcriptional activator likely downstream of the HH pathway and therefore involved in cell proliferation and differentiation [55]. 30% of BCCs have demonstrated missense mutations, mostly within the MYC box 1 domain that interacts with the tumor suppressor *FBXW7* [52]. FBXW7 functions to trigger N-MYC ubiquitin degradation [59], and mutations in the box 1 domain prevents the MYCN/FBXW7 interaction necessary for this to occur [52].

### 3.5. TERT

The role of telomerase, the product of the *TERT* gene, for immortalization of cancer cells was previously discussed. UV-signature mutations affecting the promoter region of the *TERT* gene are commonly reported in BCC, present in 39–74% [7].

### 3.6. DPH3-OXNAD1 Bidirectional Promotor

The bidirectional promotor of the *DPH3* and *OXNAD1* genes has been identified as a common site for somatic mutations in BCC, with UV-signature mutations present in 42% of tumors [60]. The *DPH3* gene is necessary to produce diphthamide, a modified histidine residue present in eukaryotic elongation factor 2, itself needed for protein synthesis. Inactivation of *DPH3* is characterized by loss of a tumor cell’s metastatic ability, and therefore the gene has a tumor suppressor activity [61].

### 3.7. Other Putative Driver Genes

Other driver genes that have been proposed as pathogenic of BCC include *PPP6C*, a gene involved in the regulation of cyclin D1 and therefore cell cycle progression, and *STK19*, a kinase of unknown function which may be involved in transcriptional regulation [7,61]. Bonilla et al. (2016) have also implicated mutations in the mitogen-associated protein kinase (MAPK) growth pathway, reporting gain of function mutations in the *KRAS, NRAS, HRAS* and *PIK3CA* genes, collectively occurring in 4% of genotyped BCCs, in addition to 4% of BCCs demonstrating mutations in ERBB2 [52]. Additional genes with identified mutations in BCCs at lower frequency include *RB1, KNSTRN, ARID1A, CASP8, RAC1* [52], *CSMD1/2, GRIN2A, PREX2* [53], and *NOTCH1/2* [52,53]. It is unclear if these genes serve as primary drivers or may represent secondary mutations in BCC development.

## 4. Targeted Therapeutics for BCC

Through this review, we have discussed our current understanding of the molecular genetics of BCC, which has been enhanced greatly through the development over the past decade of next generation sequencing technologies. While at present, surgical excision remains the gold standard therapy for BCC, alongside destructive modalities (i.e., 5-fluorouracil, imiquimod, photodynamic therapy and electrodesiccation and curettage), we are now entering a new era of molecularly targeted therapeutics, which function by modulating key molecular pathways integral to BCC pathogenesis [62].

### 4.1. HH Pathway Inhibitors

The first molecularly targeted therapeutic for BCC was the SMO inhibitor cyclopamine (see Figure 1 for site of action), but serious side effects include weight loss and death in murine models made it an nonviable therapeutic option [63]. Further drug development, however, led to the formulation of vismodegib and sonidegib, oral SMO antagonists with tolerable side effect profiles, both of which are FDA approved for the treatment of locally advanced or metastatic BCCs unsuitable for surgical excision or radiotherapy [62]. In the largest randomized controlled-trial (RCT) to date of vismodegib, STEVIE, 1215 patients with BCC were treated with 150 mg once daily. The overall response rate (ORR) according to the RECIST 1.1 criteria was reported as 68.5% for locally advanced BCC, and 36.9% for metastatic BCC. 31% of patients, however, were unable to complete treatment due to the adverse event profile, which included muscle spasms from increase intracellular calcium, alopecia from HH inhibition which is necessary for hair follicle cycling, loss of sense of taste, fatigue and weight loss [64]. An RCT of sonidegib revealed similar efficacy and adverse event profile in patients with locally advanced BCC [65]. An important issue with both of these agents, however, is the risk of resistance through acquired mutations in SMO [62]. Given the high discontinuation rates of oral SMO antagonists, a topical inhibitor, patidegib, has also been trialed with reduced disease burden in patients with Gorlin syndrome [66]. The anti-fungal agent itraconazole is additionally known to function as a weak HH pathway inhibitor, and has been suggested as a potential therapy for BCC. Early trials, however, of oral and topical itraconazole have shown mixed results [67].

### 4.2. Immune Modulators

Another potential target for BCC treatment is immunotherapy, given the anti-tumor effect of cutaneous immune cells. Cemiplimab is a monoclonal antibody which functions as an inhibitor of programmed cell death receptor 1 (PD-1), which is an immune checkpoint receptor located on lymphocytes. Upon activation by the PD-1 ligand, the checkpoint triggers down regulation of immune function and therefore immune tolerance. The PD-1 ligand has been found to be expressed on BCC cells [68], and cemiplimab has demonstrated efficacy in locally advanced and metastatic BCC [69]. Other PD-1 inhibitors, such as pembrolizumab, are also under investigation, with a proof-of-principle study of 16 patients with advanced BCCs, demonstrating an overall response rate per RECIST 1.1 of 38% after 18 weeks [70].

### 4.3. Emerging Therapeutics

Experimental therapies for BCC using novel paradigms are emerging. These include small molecular antagonists of the transcription factor downstream of the HH pathway, GLI1, which may be able to avoid the issues of resistance experienced by inhibitors acting upstream. Verteporfin is one such agent under investigation [7]. Intratumor injection of a DNAzyme targeting the mRNA of the transcription factor c-JUN, is another novel therapy that has been tested in a phase 1 study of nine patients in Australia. c-JUN expression is regulated by GLI1 and is highly expressed in BCC cells compared to normal tissue. In the trial, the agent was able to reduce c-JUN levels in all nine tumors and decrease the histological depth of five [71]. Lastly, histone deacetylase (HDAC) inhibitors may have promise in BCC, potentially through sequestering GLI to the inner nuclear membrane [72]. A phase 2 open label single arm trial of the topical HDAC inhibitor, remetinostat, demonstrated an ORR of 69.7% in 33 per-protocol tumors, alongside pathological clearance rates of 54.8% (manuscript in press; ClinicalTrials.gov Identifier NCT03180528). Acetylation of *GLI1* at the K518 locus has been shown to cause sequestration of the transcription factor inside the inner nuclear membrane, preventing its release and its associated pro-oncogenic downstream activity. HDAC isoform 1 inhibitors increase *GLI1* acetylation and through this mechanism likely suppress tumor growth [73].

### 4.4. Metastasis

Although locally destructive, BCC metastasis is exceedingly rare with an estimated frequency ranging from 1 in 1000 to 1 in 35,000 histologically identified BCCs [74]. There is a paucity of literature investigating mutations associated with metastasis, but in a small sample of eight patients, all eight patients had identical genetic mutations in both the primary tumor and metastatic site [75]. A wide variation of mutations were identified, but four of the eight patients had SMO mutations in the primary tumor or metastasis, known to provide resistance to vismodegib [75]. Greater research is needed to understand the pathogenesis of metastatic BCC.

## 5. Conclusions

The molecular basis of BCC is complex, involving an interplay of inherited genetic susceptibility and somatic mutations contributing to tumor development. Recent advances in sequencing have allowed us to better explore mutational drivers and implicated molecular pathways. The emerging therapies that we have discussed are based on these insights gained over the past decade into the molecular pathogenesis of BCC, reflecting the importance of this work. As our understanding continues to increase, we look forward to exciting, paradigm-shifting, innovation in the management of humankind’s most common cancer.

## Figures and Tables

**Table 1:** Genetic syndromes associated with inherited susceptibility to BCC, with causative gene mutations and associated molecular pathways.

Syndrome	Gene(s)	Gene Function(s)
Gorlin syndrome	*PTCH1, SUFU, PTCH2*	HH pathway members
Bazex-Dupré-Christol syndrome	*UBE2A, ACTRT1*	DNA repair, Regulation of cell cycle, HH pathway
Rombo syndrome	*Unknown*	Unknown
Generalized follicular basaloid hamartoma syndrome	*Unknown*	Unknown
Happle-Tinschert syndrome	*Unknown*	Unknown
Muir-Torre syndrome	*MSH2, MLH1, MSH6, PMS2*	DNA mismatch repair
Brooke-Spiegler syndrome	*CYLD*	NF-κβ and EGFR pathways regulator
Cowden syndrome	*PTEN*	PI3K-AKT signaling pathway
Cartilage-hair hypoplasia	*RMRP*	Immune response
Schimmelpenning syndrome	*Unknown*	Unknown
Phacomatosis pigmentokeratotica	*Unknown*	Unknown
Xeroderma pigmentosum	*XPA-XPG, XPV, POLH*	Nucleotide excision repair
Bloom syndrome	*BLM (REC0L3)*	Chromosomal stability
Werner syndrome	*WRN (REC0L2), LMNA*	Chromosomal stability
Rothmund-Thomson syndrome	*REC0L4, C160rf57*	Chromosomal stability
Schopf-Schulz-Passarge syndrome	*WNT10A*	WNT/β-catenin signaling pathway, cell proliferation and migration
Epidermodysplasia verruciformis	*TMC6 (EVER1), TMC8 (EVER2)*	Immune response and signal transduction in the endoplasmic reticulum
Oculocutaneous albinism	*TYR, OCA2, TYRP1, SLC45A2 (MATP), SLC24A5, C10orf11, 4q24*	Melanin synthesis
Hermansky-Pudlak syndrome	*HPS1-HPS8*	Melanin synthesis

Table adapted from Choquet et al. (2020) [6].

**Table 2:** Genome-wide significant single nucleotide polymorphisms associated with modulated BCC risk.

SNP	Region of Chromosome	Locus	Alleles (Major/Minor)	Minor Allele Frequency	Odds Ratio	*p*-Value
rs57142672	1p36	*RCC2*	A/G	0.34	1.13	1.0 × 10^−23^
rs61824911	1q42	*RHOU*	A/G	0.28	1.11	1.1 × 10^−14^
rs57244888	2p24	*MYCN*	T/C	0.12	0.76	4.7 × 10^−12^
rs2080303	2q33	*ALS2CR12*	C/T	0.32	1.13	7.4 × 10^−19^
rs2116709	3p13	*FOXP1*	A/T	0.40	0.90	2.3 × 10^−17^
rs191177147	3q28	*LPP*	G/T	0.39	1.11	1.2 × 10^−14^
rs35407	5p13	*SLC45A2*	G/A	0.04	0.63	5.2 × 10^−27^
rs421284	5p15	*CLPTM1L*	T/C	0.44	0.90	1.1 × 10^−18^
rs1050529	6p21.33	*HLA-B*	C/T	0.25	0.90	2.6 × 10^−9^
rs9267650	6p21.33	*NEU1*	A/T	0.05	1.17	1.1 × 10^−8^
rs9275642	6p21.32	*HLA-DQA2*	C/T	0.21	0.89	2.4 × 10^−12^
rs2294214	6p22	*CASC15*	A/C	0.32	1.07	3.1 × 10^−8^
rs12203592	6p25.3	*IRF4*	C/T	0.17	1.48	2.4 × 10^−152^
rs12210050	6p25.3	*EXOC2*	C/T	0.17	1.25	1.0 × 10^−51^
rs4710154	6q27	*MIR3939*	A/T	0.32	1.08	1.1 × 10^−8^
rs7776701	7p12	*TNS3*	C/T	0.48	0.94	4.2 × 10^−8^
rs73183643	7q22	*CUX1*	G/A	0.24	0.90	1.5 × 10^−13^
rs157935	7q32	*KLF14*	T/G	0.29	0.81	8.5 × 10^−11^
rs10093547	8q21.11	*ZFHX4*	T/G	0.06	0.82	4.6 × 10^−15^
rs11993814	8q21.13	*ZBTB10*	C/T	0.26	0.92	8.8 × 10^−11^
rs141115006	8q22	*RGS22*	C/T	0.17	0.88	2.0 × 10^−15^
rs7874604	9p21	*CDKN2B-AS1*	T/C	0.46	0.91	4.5 × 10^−13^
rs10810657	9p22	*BNC2*	A/T	0.41	0.90	1.5 × 10^−17^
rs73635312	10p14	*GATA3*	G/A	0.14	0.84	2.8 × 10^−23^
rs7907606	10q24	*OBFC1*	T/G	0.17	1.10	4.7 × 10^−9^
rs1126809	11q14	*TYR*	G/A	0.28	1.12	2.5 × 10^−19^
rs11170164	12q13	*KRT5*	C/T	0.08	1.19	1.1 × 10^−15^
rs7335046	13q32	*UBAC2*	C/G	0.12	1.26	2.9 × 10^−8^
2916300	15q13	*OCA2/HERC2*	T/C	0.29	0.87	8.2 × 10^−17^
rs1805007	16q24	*MC1R*	C/T	0.07	1.40	2.5 × 10^−63^
rs78378222	17p13	*TP53*	T/G	0.01	1.41	1.8 × 10^−10^
rs10425559	19p13	*PLIN3*	G/A	0.40	0.93	2.8 × 10^−8^
rs214785	20p13	*TGM3*	T/C	0.18	1.19	7.9 × 10^−33^
rs6059655	20q11	*RALY*	G/A	0.07	1.24	2.5 × 10^−26^
rs2776353	21q22	*LINC00111*	A/T	0.33	0.91	1.6 × 10^−12^

Table adapted from Choquet et al. (2020) [6].
